# Abnormal blood 25-hydroxyvitamin D in critically ill patients: prevalence, predictors, and its association with in-hospital mortality

**DOI:** 10.1186/s40001-022-00736-6

**Published:** 2022-07-06

**Authors:** Juntao Xie, Qingui Chen, Dejian He

**Affiliations:** 1 Intensive Care Unit, The First People’s Hospital of Chenzhou, Chenzhou, 423000 Hunan China; 2 Department of Medical Intensive Care Unit, The First Affiliated Hospital, Sun Yat-Sen University, Guangzhou, 510080 Guangdong China; 3 Department of Emergency, The First People’s Hospital of Chenzhou, No. 102 Luojiajing, Chenzhou, 423000 Hunan China

**Keywords:** Vitamin D, 25-Hydroxyvitamin D, Prognosis, Critical care

## Abstract

**Background:**

Abnormal vitamin D is prevalent in critical care settings, but its association with prognosis remains unclear. The study aimed to investigate the prevalence and predictors of abnormal blood 25-hydroxyvitamin D (25(OH)D), as well as its association with prognosis in critically ill patients.

**Methods:**

Patients aged ≥ 18 years who were once admitted to the intensive care units (ICUs) of the Beth Israel Deaconess Medical Center between 2008 and 2019 with at least one measurement record of blood 25(OH)D were included as study population. Baseline characteristics associated with deficient or elevated blood 25(OH)D were investigated by univariable logistic regression analysis. The association between abnormal blood 25(OH)D and hospital mortality was examined by multivariable logistic regression analysis.

**Results:**

A total of 1091 patients were included. Deficient 25(OH)D (< 30 ng/mL) was found in 790 (72.41%) patients and 17 (1.56%) were with an elevated level (> 60 ng/mL). A younger age, male, comorbid liver disease, and dialysis were risk factors of deficient blood 25(OH)D, while comorbid myocardial infarction, dementia, and rheumatic disease were protective factors evaluated by univariable logistic regression. Being admitted to cardiac vascular ICU or coronary care unit were associated with increased risk of elevated blood 25(OH)D. Patients with elevated blood 25(OH)D showed non-significantly higher hospital mortality compared to those with normal or deficient blood 25(OH)D (35.29% versus 14.44% and 14.56%, *P* = 0.058). After adjusted for potential confounding factors, elevated blood 25(OH)D was associated with increased risk of hospital mortality [odds ratio (OR) 3.80, 95% confidence interval (CI) 1.22–11.82, *P* = 0.021] when compared to those with normal blood 25(OH)D, but there was no significant association between deficient blood 25(OH)D and hospital mortality (OR 1.12, 95% CI 0.74–1.72, *P* = 0.589).

**Conclusions:**

These findings suggest deficient blood 25(OH)D was rather common in critically ill patients, but was not an independent risk factor of hospital mortality, while elevated blood 25(OH)D was associated with worse prognosis.

## Background

Vitamin D is a fat-soluble vitamin which is synthesized in the skin from 7-dehydrocholesterol after exposure to sunlight or ultraviolet light (vitamin D3, or cholecalciferol) or obtained from nutritional sources (vitamin D2, or ergocalciferol). It is then hydroxylated into 25-hydroxyvitamin D3 (25(OH)D) in the liver, and subsequently into 1,25-dihydroxyvitamin D3 (1,25(OH)2D) as is the active metabolite in the kidney [1, 2]. The physiologic functions of vitamin D have been well established, especially in calcium homeostasis and metabolism, and increasing evidence suggests it is also involved in cell growth, immune functions, and inflammation [3]. Although the definitions and criteria for determining vitamin D deficiency differed between investigations, a low vitamin D status is very common worldwide [4]. It is estimated that one billion people in the world have vitamin D deficiency (or insufficiency) [1], and the prevalence of vitamin D deficiency among adult population was reported to be 14–59%, which might be higher in Asian countries [5–7]. Meanwhile, vitamin D deficiency is observed to be associated with increased risk of mortality, several chronic diseases, and acute conditions in the general population [8].

For critically ill patients admitted to intensive care units (ICUs), high prevalence of vitamin D deficiency has also been reported by various studies, although the exact prevalence differed between studies [9–11]. However, the association between vitamin D deficiency and prognosis of critically ill patients remains unclear, since inconsistent findings were reported [9, 12–14]. The sample sizes in these available studies are usually very limited, and thus studies with larger sample sizes are warranted to examine the association, which is clinically relevant, because if vitamin D deficiency is associated with prognosis, vitamin D supplementation might benefit critically ill patients. A recent meta-analysis that included nine randomized controlled trials (1867 patients in total) which compared the efficacy of vitamin D supplementation with placebo suggested that the administration of vitamin D did not provide additional advantages over placebo for critically ill patients [15]. This finding is inconsistent with the conclusion from an earlier meta-analysis (including seven trials with 716 patients) that vitamin D supplementation was associated with a reduction in mortality in critically ill patients [16], in which a recent large-scale trial of more than 1000 patients with the findings that early administration of high-dose enteral vitamin D3 did not provide survival benefit was not included [17].

To further provide evidence about this topic (i.e., abnormal vitamin D in critically ill patients), we therefore conducted a study with a relatively large sample size, aiming to investigate the prevalence and predictors of abnormal blood 25(OH)D as well as its association with prognosis in critically ill patients.

## Methods

### Data source

We used data accessed from Medical Information Mart for Intensive Care IV (MIMIC IV, version 0.4) [18], which contains clinical information (including vital signs, laboratory measurements, diagnosis, administered medications) collected during hospitalizations for patients admitted to ICUs of the Beth Israel Deaconess Medical Center (i.e., a tertiary academic medical center in the United States) between 2008 and 2019. As previously described [19], in the study we used codes from the code repository mimic-iv (https://github.com/MIT-LCP/mimic-iv) for data extraction.

The database is released under the Health Insurance Portability and Accountability Act (HIPAA) safe harbor provision. Access to the database was approved by the institutional review boards (IRB) of both Beth Israel Deaconess Medical Center and Massachusetts Institute of Technology Affiliates, after completing the Collaborative Institutional Training Initiative (CITI) “Data or Specimens Only Research” course. After we consulted the IRB of The First People’s Hospital of Chenzhou, this study was exempt from further approval and patient consent due to the retrospective design, lack of direct patient intervention, and the security schema for the re-identification risk, which made the study do not meet the definition of “human subjects” research requiring IRB review. The study complied with the Declaration of Helsinki.

### Study population

**Fig. 1:**
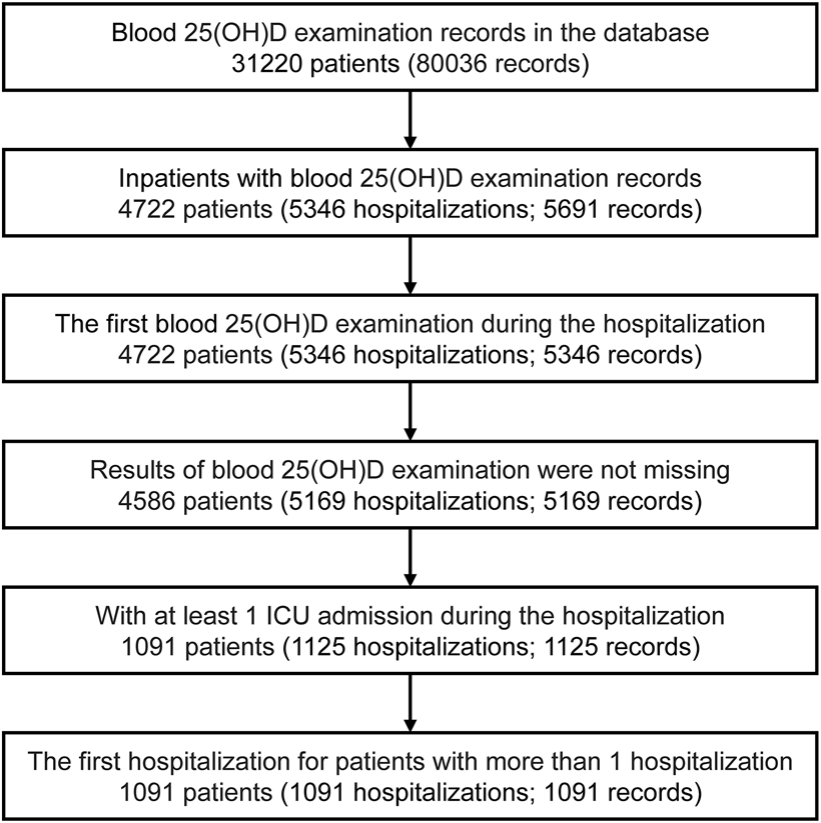
Selection of the study population. *ICU* intensive care unit

Patients aged ≥ 18 years who were once admitted to the ICUs of the Beth Israel Deaconess Medical Center between 2008 and 2019 with at least one measurement record of blood 25(OH)D during a hospitalization were included as the study population. In detail, patients included into the study should meet the below inclusion criteria: (1) patients with blood 25(OH)D measurement record(s) in the database; (2) blood 25(OH)D was measured during a hospitalization; (3) during the hospitalization, the patients were admitted to ICU(s) at least once. Patients with blood 25(OH)D measurement records but missing results were excluded. If a patient had more than one blood 25(OH)D measurement record during a hospitalization, we only used the earliest blood 25(OH)D measurement record; if a patient had more than one hospitalization that met the above inclusion criteria, we only included the earliest hospitalization. Figure 1 presents the selection of the study population.

### Levels of blood 25(OH)D

In the database, there was no information about how blood 25(OH)D was measured and which type of specimen (i.e., serum or plasma) was used for the measurement, but the type of examination (i.e., routine or urgent) was indicted in the database. The reference range of blood 25(OH)D in the database was 30-60 ng/mL. When investigating the prevalence of abnormal blood 25(OH)D and its association with prognosis in critically ill patients, we categorized levels of blood 25(OH)D as normal (30–60 ng/mL), deficient (< 30 ng/mL), and elevated (> 60 ng/mL). When exploring predictors of abnormal blood 25(OH)D, we categorized levels of blood 25(OH)D as deficient (< 30 ng/mL) and nondeficient (≥ 30 ng/mL), or elevated (> 60 ng/mL) and non-elevated (≤ 60 ng/mL).

### Baseline characteristics and prognosis

In the study the below variables were studied as baseline characteristics: age, sex, marital status, ethnicity, admission location, type of intensive care unit, Glasgow Coma Scale, Sequential Organ Failure Assessment (SOFA) [20], Simplified Acute Physiology Score II (SAPS II) [21], Charlson Comorbidity Index [22], and specific comorbidities including myocardial infarction, congestive heart failure, peripheral vascular disease, chronic pulmonary disease, mild liver disease, severe liver disease, renal disease, dialysis, diabetes without complications, diabetes with complications, cerebrovascular disease, dementia, paraplegia, rheumatic disease, malignant tumor, and acquired immunodeficiency syndrome. The study outcomes were hospital mortality, and the length of hospital stay.

### Statistical analysis

Results were presented as mean ± standard deviation or median (25–75th percentile) for continuous variables, and number (percentage) for categorical variables. Comparisons between groups were examined by one-way ANOVA or Kruskal–Wallis H test for continuous variables and Chi-squared test or Fisher’s exact test for categorical variables. The associations between baseline characteristics and abnormal blood 25(OH)D (i.e., deficient (< 30 ng/mL) versus nondeficient (≥ 30 ng/mL); or elevated (> 60 ng/mL) versus non-elevated (≤ 60 ng/mL)) were examined by univariable logistic regression analyses. The association between abnormal blood 25(OH)D (i.e., deficient, elevated, versus normal) and hospital mortality was examined by multivariable logistic regression analysis. In addition to a crude model, the below models were employed: Model 1 = blood 25(OH)D (i.e., < 30 ng/mL, 30–60 ng/mL, > 60 ng/mL) + age + sex + SAPS II + Charlson Comorbidity Index; Model 2 = blood 25(OH)D (i.e., < 30 ng/mL, 30–60 ng/mL, > 60 ng/mL) + age + sex + SAPS II + Charlson Comorbidity Index + type of examination + type of intensive care unit + dialysis. A P value less than 0.05 was considered to indicate statistical significance. Empower(R) (www.empowerstats.com; X&Y solutions, Inc., Boston, MA, USA) and R software, version 3.4.3 (http://www.r-project.org; R Foundation for Statistical Computing, Vienna, Austria) were used for statistical analyses.

## Results

### Prevalence of abnormal blood 25(OH)D

A total of 1091 patients were included (Fig. 1) with a mean age of 61.47 ± 15.70 years. Half (50.69%) of the patients were male, and the majority was White (61.50%). Most of the patients were admitted via emergency room (43.08%) or from other hospitals (29.51%). Medical intensive care unit (34.74%) and medical/surgical intensive care unit (22.09%) were the most frequent ICUs that the patients admitted to. The median SOFA of the patients was 6 (4–10), and the mean SAPS II was 39.26 ± 13.84. Renal disease (34.65%), congestive heart failure (32.45%), mild liver disease (27.13%), chronic pulmonary disease (24.66%), diabetes without complications (24.20%), and severe liver disease (19.89%) were the most prevalent among the studied comorbidities.

Among the study population, 26.03% were with normal blood 25(OH)D (38.91 ± 7.57 ng/mL), 72.41% were with deficient blood 25(OH)D (16.73 ± 6.76 ng/mL), and 1.56% were with elevated blood 25(OH)D (76.18 ± 16.07 ng/mL). As presented in Table 1, there were some differences in baseline characteristics between patients with different levels of blood 25(OH)D. Compared to patients with normal blood 25(OH)D, patients with deficient blood 25(OH)D had a younger age (59.69 ± 15.59 versus 66.09 ± 15.14 years), a higher proportion of male sex (53.67 versus 43.31%), higher prevalence of comorbid mild liver disease (29.87% versus 19.01%), severe liver disease (21.77% versus 14.79%), and dialysis (16.58% versus 10.56%), but lower prevalence of comorbid myocardial infarction (13.04% versus 17.96%), dementia (3.54% versus 6.34%), and rheumatic disease (3.67% versus 7.04%). While for patients with elevated blood 25(OH)D, they appeared to be more likely to admit to cardiac vascular ICU (17.65% versus 6.34%) or coronary care unit (17.65% versus 9.86%) when compared to those with normal blood 25(OH)D.

**Table 1:** Baseline characteristics of the study population

	Overall	Normal (30–60 ng/mL)	Deficient (< 30 ng/mL)	Elevated (> 60 ng/mL)	*P* value
	(*n* = 1091, 100%)	(*n* = 284, 26.03%)	(*n* = 790, 72.41%)	(*n* = 17, 1.56%)	
Blood 25-hydroxyvitamin D (ng/mL)	23.43 ± 13.79	38.91 ± 7.57	16.73 ± 6.76	76.18 ± 16.07	< 0.001
Type of examination					
Routine	597 (54.72%)	163 (57.39%)	424 (53.67%)	10 (58.82%)	0.526
Urgent	494 (45.28%)	121 (42.61%)	366 (46.33%)	7 (41.18%)	
Age (years)	61.47 ± 15.70	66.09 ± 15.14	59.69 ± 15.59	67.06 ± 13.34	< 0.001
Sex					
Male	553 (50.69%)	123 (43.31%)	424 (53.67%)	6 (35.29%)	0.005
Female	538 (49.31%)	161 (56.69%)	366 (46.33%)	11 (64.71%)	
Marital status					
Single	383 (35.11%)	82 (28.87%)	298 (37.72%)	3 (17.65%)	0.009
Married	420 (38.50%)	111 (39.08%)	301 (38.10%)	8 (47.06%)	
Divorced	87 (7.97%)	24 (8.45%)	62 (7.85%)	1 (5.88%)	
Widowed	113 (10.36%)	44 (15.49%)	65 (8.23%)	4 (23.53%)	
Unknown	88 (8.07%)	23 (8.10%)	64 (8.10%)	1 (5.88%)	
Ethnicity					
White	671 (61.50%)	192 (67.61%)	466 (58.99%)	13 (76.47%)	0.036
Black/African American	157 (14.39%)	27 (9.51%)	127 (16.08%)	3 (17.65%)	
Hispanic/Latino	44 (4.03%)	8 (2.82%)	36 (4.56%)	0 (0.00%)	
Asian	39 (3.57%)	6 (2.11%)	33 (4.18%)	0 (0.00%)	
Other/unknown	180 (16.50%)	51 (17.96%)	128 (16.20%)	1 (5.88%)	
Admission location					
Emergency room	470 (43.08%)	142 (50.00%)	320 (40.51%)	8 (47.06%)	0.051
Physician referral	156 (14.30%)	37 (13.03%)	117 (14.81%)	2 (11.76%)	
Transfer from hospital	322 (29.51%)	79 (27.82%)	238 (30.13%)	5 (29.41%)	
Walk-in/self-referral	67 (6.14%)	11 (3.87%)	56 (7.09%)	0 (0.00%)	
Procedure site	5 (0.46%)	1 (0.35%)	4 (0.51%)	0 (0.00%)	
Transfer from skilled nursing facility	23 (2.11%)	4 (1.41%)	19 (2.41%)	0 (0.00%)	
Clinic referral	34 (3.12%)	9 (3.17%)	24 (3.04%)	1 (5.88%)	
Post-anesthesia care unit	4 (0.37%)	0 (0.00%)	3 (0.38%)	1 (5.88%)	
Information not available	8 (0.73%)	1 (0.35%)	7 (0.89%)	0 (0.00%)	
Ambulatory surgery transfer	2 (0.18%)	0 (0.00%)	2 (0.25%)	0 (0.00%)	
Type of intensive care unit					
Medical Intensive Care Unit	379 (34.74%)	97 (34.15%)	280 (35.44%)	2 (11.76%)	0.154
Medical/Surgical Intensive Care Unit	241 (22.09%)	62 (21.83%)	175 (22.15%)	4 (23.53%)	
Cardiac Vascular Intensive Care Unit	55 (5.04%)	18 (6.34%)	34 (4.30%)	3 (17.65%)	
Surgical Intensive Care Unit	181 (16.59%)	39 (13.73%)	139 (17.59%)	3 (17.65%)	
Trauma Surgical Intensive Care Unit	102 (9.35%)	25 (8.80%)	75 (9.49%)	2 (11.76%)	
Coronary Care Unit	89 (8.16%)	28 (9.86%)	58 (7.34%)	3 (17.65%)	
Neuro Surgical Intensive Care Unit	25 (2.29%)	9 (3.17%)	16 (2.03%)	0 (0.00%)	
Neuro Intermediate	13 (1.19%)	2 (0.70%)	11 (1.39%)	0 (0.00%)	
Neuro Stepdown	5 (0.46%)	3 (1.06%)	2 (0.25%)	0 (0.00%)	
Post-anesthesia Care Unit	1 (0.09%)	1 (0.35%)	0 (0.00%)	0 (0.00%)	
Glasgow Coma Scale	14 (10–15)	14 (10–15)	14 (10–15)	13 (11–15)	0.748
SOFA	6 (4–10)	6 (3.75–9)	6 (4–10)	6 (4–11)	0.528
SAPS II	39.26 ± 13.84	39.80 ± 13.90	39.02 ± 13.87	41.76 ± 11.58	0.541
Charlson Comorbidity Index	6 (4–8)	6 (4–8)	6 (4–8)	6 (6–8)	0.115
Myocardial infarction	158 (14.48%)	51 (17.96%)	103 (13.04%)	4 (23.53%)	0.073
Congestive heart failure	354 (32.45%)	106 (37.32%)	245 (31.01%)	3 (17.65%)	0.063
Peripheral vascular disease	106 (9.72%)	25 (8.80%)	79 (10.00%)	2 (11.76%)	0.809
Chronic pulmonary disease	269 (24.66%)	75 (26.41%)	189 (23.92%)	5 (29.41%)	0.636
Mild liver disease	296 (27.13%)	54 (19.01%)	236 (29.87%)	6 (35.29%)	0.001
Severe liver disease	217 (19.89%)	42 (14.79%)	172 (21.77%)	3 (17.65%)	0.040
Renal disease	378 (34.65%)	106 (37.32%)	263 (33.29%)	9 (52.94%)	0.132
Dialysis	164 (15.03%)	30 (10.56%)	131 (16.58%)	3 (17.65%)	0.049
Diabetes without complications	264 (24.20%)	62 (21.83%)	199 (25.19%)	3 (17.65%)	0.430
Diabetes with complications	178 (16.32%)	37 (13.03%)	137 (17.34%)	4 (23.53%)	0.173
Cerebrovascular disease	119 (10.91%)	36 (12.68%)	81 (10.25%)	2 (11.76%)	0.529
Dementia	47 (4.31%)	18 (6.34%)	28 (3.54%)	1 (5.88%)	0.131
Paraplegia	43 (3.94%)	13 (4.58%)	29 (3.67%)	1 (5.88%)	0.731
Rheumatic disease	49 (4.49%)	20 (7.04%)	29 (3.67%)	0 (0.00%)	0.042
Malignant tumor	183 (16.77%)	41 (14.44%)	138 (17.47%)	4 (23.53%)	0.379
Acquired immunodeficiency syndrome	14 (1.28%)	4 (1.41%)	10 (1.27%)	0 (0.00%)	0.879

*SOFA* Sequential Organ Failure Assessment, *SAPS II* Simplified Acute Physiology Score II

### Predictors of abnormal blood 25(OH)D

As presented in Table 2, a younger age (odds ratio (OR) 0.97, 95% confidence interval (CI) 0.96–0.98 per 1-year increase), male (OR 0.65, 95% CI 0.50–0.85), comorbid mild liver disease (OR 1.71, 95% CI 1.24–2.36), severe liver disease (OR 1.58, 95% CI 1.11–2.27), and dialysis (OR 1.61, 95% CI 1.07–2.43) were risk factors of deficient blood 25(OH)D, while comorbid myocardial infarction (OR 0.67, 95% CI 0.47–0.96), dementia (OR 0.55, 95% CI 0.30–0.99), and rheumatic disease (OR 0.54, 95% CI 0.30–0.96) were protective factors. As presented in Table 3, being admitted to cardiac vascular ICU (OR 10.87, 95% CI 1.78–66.62, versus being admitted to medical ICU) or coronary care unit (OR 6.58, 95% CI 1.08–39.96, versus being admitted to medical ICU) were associated with increased risk of elevated blood 25(OH)D.

**Table 2:** Baseline characteristics associated with deficient blood 25-hydroxyvitamin D (< 30 ng/mL versus ≥ 30 ng/mL) by univariable logistic regression

	No. patients	No. patients with 25(OH)D < 30 ng/mL	Odds ratio	95% CI	*P* value
Age (years)	1091	790	0.97	0.96–0.98	< 0.001
Sex					
Male	553	424	1 (Reference)		
Female	538	366	0.65	0.50–0.85	0.002
Marital status					
Single	383	298	1 (Reference)		
Married	420	301	0.72	0.52–0.99	0.046
Divorced	87	62	0.71	0.42–1.19	0.195
Widowed	113	65	0.39	0.25–0.60	< 0.001
Unknown	88	64	0.76	0.45–1.29	0.309
Ethnicity					
White	671	466	1 (Reference)		
Black/African American	157	127	1.86	1.21–2.86	0.005
Hispanic/Latino	44	36	1.98	0.90–4.33	0.088
Asian	39	33	2.42	1.00–5.86	0.050
Other/unknown	180	128	1.08	0.75–1.55	0.666
Type of intensive care unit					
Medical Intensive Care Unit	379	280	1 (Reference)		
Medical/Surgical Intensive Care Unit	241	175	0.94	0.65–1.35	0.728
Cardiac Vascular Intensive Care Unit	55	34	0.57	0.32–1.03	0.064
Surgical Intensive Care Unit	181	139	1.17	0.77–1.77	0.457
Trauma Surgical Intensive Care Unit	102	75	0.98	0.60–1.61	0.943
Coronary Care Unit	89	58	0.66	0.40–1.08	0.100
Neuro Surgical Intensive Care Unit	25	16	0.63	0.27–1.47	0.283
Neuro Intermediate	13	11	1.94	0.42–8.93	0.392
Neuro Stepdown	5	2	0.24	0.04–1.43	0.116
Post-anesthesia Care Unit	1	0	–	–	–
Glasgow Coma Scale	1091	790	1.00	0.96–1.04	0.921
SOFA	1091	790	1.02	0.99–1.05	0.248
SAPS II	1091	790	1.00	0.99–1.00	0.342
Charlson Comorbidity Index	1091	790	0.96	0.92–1.00	0.078
Comorbidities^$^					
Myocardial infarction	158	103	0.67	0.47–0.96	0.029
Congestive heart failure	354	245	0.79	0.60–1.05	0.101
Peripheral vascular disease	106	79	1.13	0.71–1.78	0.608
Chronic pulmonary disease	269	189	0.87	0.64–1.18	0.364
Mild liver disease	296	236	1.71	1.24–2.36	0.001
Severe liver disease	217	172	1.58	1.11–2.27	0.012
Renal disease	378	263	0.81	0.61–1.06	0.128
Dialysis	164	131	1.61	1.07–2.43	0.021
Diabetes without complications	264	199	1.22	0.89–1.68	0.216
Diabetes with complications	178	137	1.33	0.91–1.94	0.138
Cerebrovascular disease	119	81	0.79	0.52–1.19	0.262
Dementia	47	28	0.55	0.30–0.99	0.047
Paraplegia	43	29	0.78	0.41–1.50	0.458
Rheumatic disease	49	29	0.54	0.30–0.96	0.037
Malignant tumor	183	138	1.20	0.83–1.74	0.320
Acquired immunodeficiency syndrome	14	10	0.95	0.30–3.06	0.934

*CI* confidence interval, *SOFA* Sequential Organ Failure Assessment, *SAPS II*, Simplified Acute Physiology Score II

^$^Versus patients without the commodities

**Table 3:** Baseline characteristics associated with elevated blood 25-hydroxyvitamin D (> 60 ng/mL versus ≤ 60 ng/mL) by univariable logistic regression

	No. patients	No. patients with 25(OH)D > 60 ng/mL	Odds ratio	95% CI	*P* value
Age (years)	1091	17	1.03	0.99–1.06	0.141
Sex					
Male	553	6	1 (Reference)		
Female	538	11	1.90	0.70–5.18	0.208
Marital status					
Single	383	3	1 (Reference)		
Married	420	8	2.46	0.65–9.34	0.186
Divorced	87	1	1.47	0.15–14.33	0.739
Widowed	113	4	4.65	1.02–21.08	0.046
Unknown	88	1	1.46	0.15–14.17	0.746
Ethnicity			2.46	0.65–9.34	0.186
White	671	13	1 (Reference)		
Black/African American	157	3	0.99	0.28–3.50	0.983
Hispanic/Latino	44	0	-	-	-
Asian	39	0	-	-	-
Other/Unknown	180	1	0.28	0.04–2.18	0.225
Type of intensive care unit					
Medical Intensive Care Unit	379	2	1 (Reference)		
Medical/Surgical Intensive Care Unit	241	4	3.18	0.58–17.51	0.183
Cardiac Vascular Intensive Care Unit	55	3	10.87	1.78–66.62	0.010
Surgical Intensive Care Unit	181	3	3.18	0.53–19.18	0.208
Trauma Surgical Intensive Care Unit	102	2	3.77	0.52–27.10	0.187
Coronary Care Unit	89	3	6.58	1.08–39.96	0.041
Neuro Surgical Intensive Care Unit	25	0	–	–	–
Neuro Intermediate	13	0	–	–	–
Neuro Stepdown	5	0	–	–	–
Post-anesthesia Care Unit	1	0	–	–	–
Glasgow Coma Scale	1091	17	1.00	0.88–1.15	0.950
SOFA	1091	17	1.02	0.92–1.13	0.741
SAPS II	1091	17	1.01	0.98–1.05	0.452
Charlson Comorbidity Index	1091	17	1.09	0.94–1.27	0.238
Comorbidities^$^					
Myocardial infarction	158	4	1.84	0.59–5.71	0.293
Congestive heart failure	354	3	0.44	0.13–1.55	0.201
Peripheral vascular disease	106	2	1.24	0.28–5.51	0.774
Chronic pulmonary disease	269	5	1.28	0.45–3.66	0.647
Mild liver disease	296	6	1.47	0.54–4.02	0.448
Severe liver disease	217	3	0.86	0.25–3.02	0.816
Renal disease	378	9	2.15	0.82–5.62	0.119
Dialysis	164	3	1.22	0.35–4.28	0.761
Diabetes without complications	264	3	0.67	0.19–2.34	0.528
Diabetes with complications	178	4	1.59	0.51–4.94	0.421
Cerebrovascular disease	119	2	1.09	0.25–4.83	0.909
Dementia	47	1	1.40	0.18–10.76	0.748
Paraplegia	43	1	1.54	0.20–11.85	0.681
Rheumatic disease	49	0	–	–	–
Malignant tumor	183	4	1.54	0.50–4.77	0.456
Acquired immunodeficiency syndrome	14	0	–	–	–

*CI* confidence interval, *SOFA* Sequential Organ Failure Assessment, *SAPS II* Simplified Acute Physiology Score II

^$^Versus patients without the comorbidities

### Association of abnormal blood 25(OH)D with prognosis

The overall hospital mortality was 14.85% among the study population with a median length of hospital stay of 17.39 (8.88–32.78) days. As presented in Table 4, similar hospital mortality rates and lengths of hospital stay were observed between patients with normal and deficient blood 25(OH)D, while patients with elevated blood 25(OH)D showed higher hospital mortality (35.29% versus about 14%) and longer length of hospital stay (median 24.82 days versus about 17 days). As presented in Table 5, after adjusted for potential confounding factors, elevated blood 25(OH)D was associated with increased risk of hospital mortality (Model 1: OR 3.25, 95% CI 1.07–9.89; Model 2: OR 3.80, 95% CI 1.22–11.82) when compared to those with normal blood 25(OH)D, but there was no significant association between deficient blood 25(OH)D and hospital mortality (Model 1: OR 1.15, 95% CI 0.76–1.75; Model 2: OR 1.12, 95% CI 0.74–1.72).

**Table 4:** Prognosis of the study population

	Overall	Normal (30–60 ng/mL)	Deficient (< 30 ng/mL)	Elevated (> 60 ng/mL)	*P* value
	(*n* = 1091)	(*n* = 284)	(*n* = 790)	(*n* = 17)	
Hospital mortality	162 (14.85%)	41 (14.44%)	115 (14.56%)	6 (35.29%)	0.058
Length of hospital stay (days)	17.39 (8.88–32.78)	16.73 (8.65–28.29)	17.86 (8.96–33.89)	24.82 (12.73–44.77)	0.160

**Table 5:** Association between levels of blood 25-hydroxyvitamin D and hospital mortality

Blood 25-hydroxyvitamin D	Odds ratio	95% CI	*P* value
Crude			
Normal (30–60 ng/mL)	1 (Reference)		
Deficient (< 30 ng/mL)	1.01	0.69–1.48	0.961
Elevated (> 60 ng/mL)	3.23	1.13–9.22	0.028
Adjusted (model 1)			
Normal (30–60 ng/mL)	1 (Reference)		
Deficient (< 30 ng/mL)	1.15	0.76–1.75	0.501
Elevated (> 60 ng/mL)	3.25	1.07–9.89	0.038
Adjusted (model 2)			
Normal (30–60 ng/mL)	1 (Reference)		
Deficient (< 30 ng/mL)	1.12	0.74–1.72	0.589
Elevated (> 60 ng/mL)	3.80	1.22–11.82	0.021

Model 1 was adjusted for age, sex, SAPS II, and Charlson Comorbidity Index; Model 2 was adjusted for Model 1, plus type of examination, type of intensive care unit, and dialysis

*CI*, confidence interval, *SAPS II* Simplified Acute Physiology Score II

## Discussion

By including a relatively large cohort of ICU patients (about 1000, which may be the largest one so far), we investigated the prevalence and predictors of abnormal blood 25(OH)D as well as its association with prognosis in critically ill patients. The main findings of our study are: (1) deficient blood 25(OH)D is rather common (about 70%) in ICU patients; (2) some patient profiles are associated with increased risk of deficient blood 25(OH)D, such as liver diseases and receiving dialysis; (3) after taking potential confounding factors into account, deficient blood 25(OH)D is not associated with increased risk of hospital mortality; instead, elevated blood 25(OH)D is an independent risk factor of hospital mortality. Given the findings from currently available studies are inconsistent, our findings derived from a large cohort provide new evidence for this topic. Although deficient blood 25(OH)D is very prevalent in ICU patients, our study suggests it is not associated with prognosis, and therefore efforts should be put into the management of other conditions which may be the reasons for deficient blood 25(OH)D, instead of hoping to improve prognosis of ICU patients simply by vitamin D supplementation.

Abnormal blood 25(OH)D in critically ill patients and its association with prognosis has been evaluated in several studies. Venkatram et al. [23] investigated 437 patients admitted to a medical ICU and found 77.8% of the patients were with 25(OH)D deficiency (i.e., 0–19.9 ng/dL) and 16.9% were with 25(OH)D insufficiency (i.e., 20–29.9 ng/dL), and patients with a decreased level of 25(OH)D tended to be younger, male, and with kidney disease. They found 25(OH)D deficiency, but not 25(OH)D insufficiency, was associated with increased risk of hospital mortality. Mayr et al. [24] investigated 176 critically ill patients and found 55% patients had a severe deficiency (defined as 25(OH)D < 10 ng/mL) and 23% had moderate deficiency (defined as 25(OH)D 10–19 ng/mL). A severe deficiency with levels < 10 ng/mL is a risk factor for increased mortality, especially in patients with cirrhosis. These studies support deficient 25(OH)D as a risk factor of mortality. However, Higgins et al. [25] included 196 medical/surgical ICU patients and found low 25(OH)D was not significantly associated with 28-day all-cause mortality, but was associated with longer time to ICU discharge alive and a trend toward increased risk of ICU-acquired infection. Jevalikar et al. [26] investigated 410 hospitalized patients with COVID-19, of which 48.2% were with vitamin D deficiency (defined as serum 25(OH)D level < 20 ng/mL), and found serum 25(OH)D levels at admission did not correlate with clinical outcomes, and receiving cholecalciferol did not make any difference to the outcomes. Similar results were also reported by Gomes et al. [27], Maamer et al. [28], and Aygencel et al. [29]. From these available studies, it could be observed that the prevalence of deficient blood 25(OH)D varied, which might be due to different criteria for determining deficient 25(OH)D and different study populations. General speaking, the prevalence is rather high in these studies, which is consistent with our study.

About the risk factors for deficient 25(OH)D, our findings are also similar to studies that investigated patient profiles associated with deficient 25(OH)D. These findings might be more important in clinical practice, which could be the reasons for abnormal 25(OH)D and warrant interventions, such as liver or renal diseases, since liver and kidney are the two organs involved in the metabolism of vitamin D [1, 2]. The inconsistent findings about the association between deficient 25(OH)D and prognosis between studies may be due to the very limited sample sizes, which could not provide enough statistical power, or the selected study population. The large sample size and a mixed ICU population relieve this concern in our study, which is the main strength of our findings. In the study, we also investigated the prevalence of elevated blood 25(OH)D and its association with prognosis, and we found elevated blood 25(OH)D is significantly associated with hospital mortality. Unlike investigations on deficient 25(OH)D, evidence about elevated 25(OH)D in ICU patients is rather limited. Our findings about this provide some insights, but it should be interpreted cautiously, since the sample size of this subgroup is very limited (*n* = 17).

Some limitations in our study should be noted. First, due to a retrospective study design and limitation of the data source, the exact measurement method of blood 25(OH)D is unknown in our study. In addition, we only investigated blood 25(OH)D measured at a single time point during a hospitalization, instead repeated measurements. This could be an issue, since a single random measurement may not be reflective of the vitamin D status in ICU patients because of changes in fluid administration, and intra-day variation in 25(OH)D levels [30]. Second, the data used in our study are from a single center, and therefore our findings may lack generalizability. Third, the study population was admitted to ICUs between 2008 and 2019, and patient profiles and interventions may change greatly during such a long period. Due to the exact years of admission were blinded in the data, a subgroup analysis was impossible. Last, given the nature of an observational study, residual confounding cannot be ruled out in the association of abnormal blood 25(OH)D with prognosis.

## Conclusions

Deficient blood 25(OH)D was rather common in critically ill patients, but was not an independent risk factor of hospital mortality, while elevated blood 25(OH)D was associated with worse prognosis.

## Data Availability

The data that support the findings of this study are available from PhysioNet but restrictions apply to the availability of these data, which were used under license for the current study, and so are not publicly available. Data are, however, available to access after completing the required procedures of requesting access to the database (see https://physionet.org/content/mimiciv/0.4/).

## Abbreviations

25(OH)D: 25-Hydroxyvitamin D3;
1,25(OH)2D: 1,25-Dihydroxyvitamin D3;
ICUs: Intensive care units;
MIMIC IV: Medical Information Mart for Intensive Care IV;
HIPAA: Health Insurance Portability and Accountability Act;
IRB: Institutional review boards;
CITI: Collaborative Institutional Training Initiative;
SOFA: Sequential Organ Failure Assessment;
SAPS II: Simplified Acute Physiology Score II;
OR: Odds ratio;
CI: Confidence interval
